# *Drosophila melanogaster* as a model organism to investigate sex specific differences

**DOI:** 10.1038/s41598-025-04497-0

**Published:** 2025-06-04

**Authors:** Sonja Dähn, Anika E. Wagner

**Affiliations:** 1 Institute of Nutritional Science, Justus Liebig University, Wilhelmstrasse 20, 35392 Giessen, Germany; 2 Center for Sustainable Food Systems, Justus Liebig University, Senckenbergstrasse 3, 35390 Giessen, Germany

**Keywords:** *Drosophila melanogaster*, Insulin signaling, Immunity, Imd signaling, Toll signaling, Sex differences, Genetics, Immunology, Psychology, Health care

## Abstract

Sex differences in physiology, anatomy, behavior, and genetics are well-documented throughout the animal kingdom. These differences are often neglected in research. This imbalance can have detrimental effects, as seen in cases where certain drugs have stronger side effects in females than in males. The fruit fly, *Drosophila melanogaster*, presents a promising model for studying these sex-specific differences because it shares many disease-related genes and is easy to use. RNA of 10-day-old and 30-day-old *D. melanogaster* (w^1118^) was isolated and sequenced. In 10-day-old flies 3969 genes are significantly higher expressed in males than in females, and 7176 genes are significantly lower expressed in males. In 30-day-old males 3735 genes are significantly higher expressed than in females, and 7101 genes are significantly lower expressed. In detail, the present study shows that male flies exhibit higher expression levels of genes involved in toll signaling, Imd signaling, insulin signaling, and lipid metabolism. These findings highlight *D. melanogaster* as a valuable model organism for studying sex differences in these highly conserved signaling pathways. This model could help analyzing the sex-specific effects of dietary interventions or drugs, ultimately leading to a better understanding of sex-specific interconnections and improving the development of more effective, sex-specific medical treatments.

**Supplementary Information:**

The online version contains supplementary material available at 10.1038/s41598-025-04497-0.

## Introduction

Biological sex is a fundamental variable influencing physiology, disease susceptibility, and treatment outcomes across species, including humans. Sexual dimorphism, including physiological properties, anatomy, behavior and genetics is a well-documented phenomenon in the animal kingdom^1^. Even though it is known that males and females differ in many aspects, most experimental research has predominantly involved only one sex (mainly males), without addressing sex-specific differences. For example, in 2021 Brady and colleagues analyzed 4420 SARS-CoV-2 studies^2^. The researchers found out, that only 21% addesses sex in the recruitment and only about 5.4% planed to analyze the effects in regard of sex. Alarmingly, the results were often generalized to both sexes^3^ which may result in detrimental effects on one sex. For instance, many drugs have been withdrawn due to adverse side effects in women. One example is the appetite suppressant subitramine, which has been linked to adverse cardiovascular events and psychiatric side effects, especially in women^4^. The focus on research in males does not only affect drug research, but also disease research. A common example are cardiovascular diseases (CVD) where women are typically underrepresented in clinical trials. The specific symptoms being associated with an acute myocardial infarction such as chest and arm pain are accepted as general, although women usually exhibit atypical symptoms. This may be at least one reason why women with acute coronary syndromes are less likely to receive correct treatment and therefore have a higher risk of death^5–7^. Other examples of sexually dimorphic development and manifestation of diseases are Diabetes mellitus type 2 (DMT2) and Parkinson’s disease, both of which cause more deaths in men than in women, while women die more frequently from chronic lower respiratory diseases and Alzheimer’s disease^8^. Women can store (nutritional) energy more effectively than men. In mammals females store about 10% more body fat compared to males^9^. Additionally, the distribution of the fat storages also differs between the sexes with women having more subcutaneous and men more visceral fat^10^. Consequently, there are sex differences in lipid metabolism (storage, mobilization and oxidation), but also in carbohydrate and protein metabolism^10^, suggesting that nutritional interventions are also likely to affect men and women in different ways.

It is also known that women are less often affected by infectious diseases than men. This immunity-related sex dimorphism has been assumed to be caused by differences in hormonal levels. However, the role of estrogen in immunity seems to be unclear. On the one hand, in several model organisms for chronic inflammatory diseases, estrogen seems to have a suppressive effect on immune response, on the other hand, also proinflammatory effects have been documented in some model organisms for autoimmune diseases^11^.

Although it is well known that various diseases have different incidences and forms of progression in men and women, most medical research has only been performed in men (or male model organisms) neglecting sex-specific differences. One reason may be high costs of studies using human subjects or common model organisms like laboratory rodents. Therefore, there is a great need for a cost-effective alternative model organism in which males and females can be easily compared. The fruit fly *Drosophila melanogaster* has been used as a model organism since 1905^12^, initially as a model for genetics by investigating spontaneously occurring mutations and their inheritance^13^. However, in recent years *D. melanogaster* has also gained interest as a model for various chronic human diseases (e.g. Alzheimer’s disease, DMT2), conserved biological processes, including insulin signaling, innate immunity (Imd and toll signaling), and energy metabolism (insulin signaling), many of which show marked sex-specific regulation and physiological approaches, including nutrition, due in part to cost-effective housing and short generation time^14,15^. Many disease-associated genes are highly conserved in both humans and in *D. melanogaster*, including insulin signaling, which is homologous to insulin signaling in humans. Furthermore, homologies between human and *D. melanogaster* organs can be observed: the fruit fly has a brain, a heart, a digestive system, Malpighian tubules (they have a similar function as mammalian kidneys) and also a fat body (which has similar functions to the human fat tissue and liver). Female flies store more fat than male flies (comparable to the sex specific differences in mammals), which may be due to differences in the glucagon-like Adipokinetic hormone (Akh) pathway^16^. Nutritional interventions affect males and females differently. A low-sugar diet has effects on the body size and insulin signaling in male and female larvae via sex-specific mechanisms^17^. A high-protein diet increases body size in female but not in male larvae by activating insulin signaling in females but not in males^18^. Although sex differences are also known for *D. melanogaster* most studies focus on either males or females without discussing the role of sex specific differences^19–23^. Additionally, most of the studies have used larvae, so there is a lack of data comparing adult male and female flies, also within the context of sex-specific effects of nutritional interventions.

Although it is known, that men and women differ and that these differences are reflected, for example, in the prevalence and progression of various diseases, there is still a lack of research focusing on the comparison of men and women. In the present work, we aimed to show that the fruit fly *D. melanogaster* can be used as a comparatively inexpensive and easy-to-handle model to study sex-specific differences. Therefore, we investigated the gene expression of highly conserved signaling pathways, involving Imd and toll signaling, as well as insulin signaling. We focused on comparing untreated mated male and female flies to reveal sex specific differences in gene expression at a basal level. We sequenced 10- and 30-day-old male and female flies and compared those signaling pathways that orchestrate energy metabolism and immune function.

## Results

**Fig. 1:**
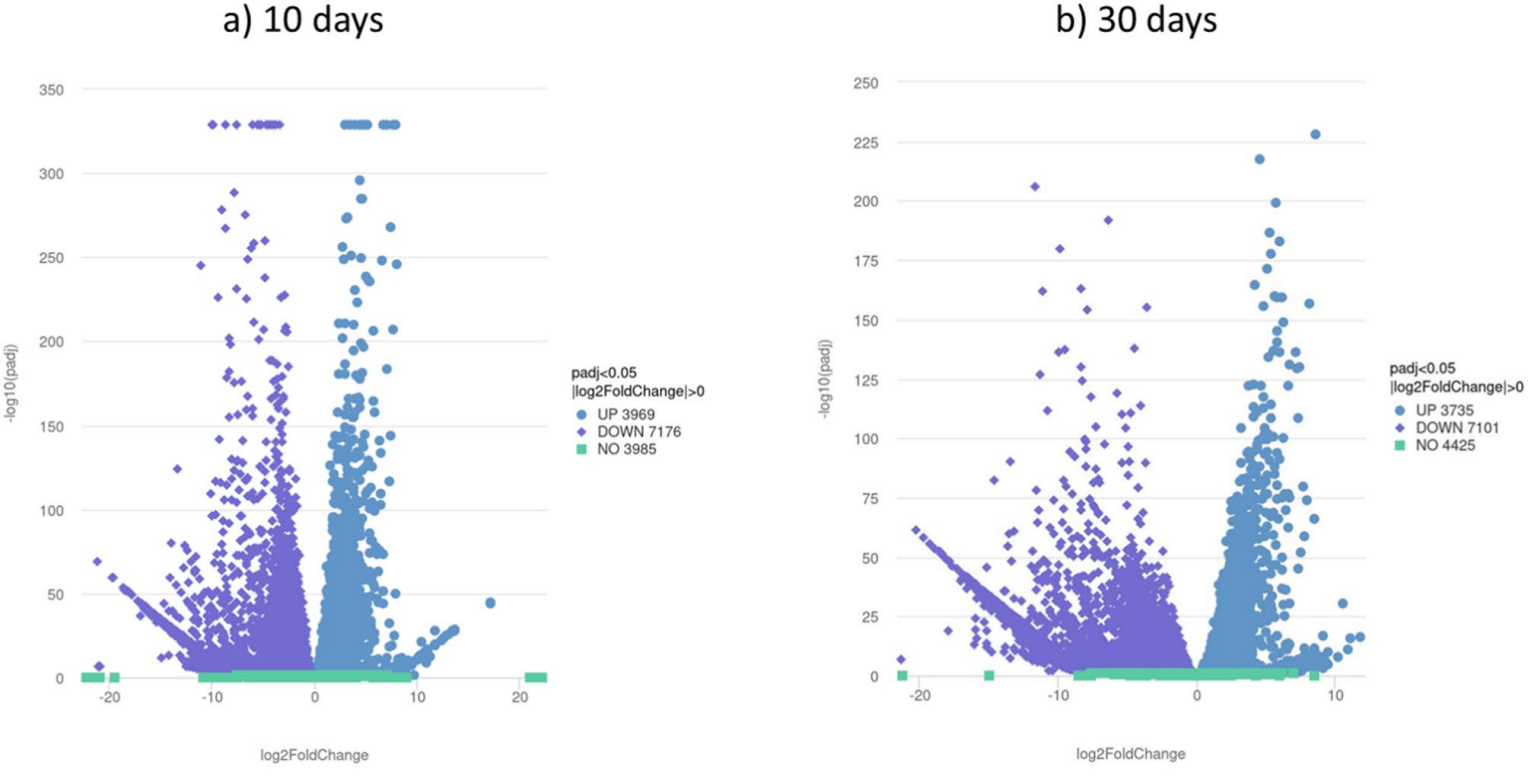
Volcano plot showing the number of differentially expressed genes between 10-day-old (**a**) and 30-day-old (**b**) female and male flies. Genes that are higher expressed in males are spotted in blue, genes that are higher expressed in females are spotted in purple, genes that showed no significant difference in expression are spotted in green.

Sequencing of the mRNA of 10-day-old flies revealed that in female flies a total number of 12274 genes was detected, whereas in male flies 14734 genes were detected. 3969 genes are significantly higher expressed in males than in females, and 7176 genes are significantly lower expressed in males (Fig. 1 a)).

Sequencing of the mRNA of 30-day-old flies revealed that in males a total number of 14112 genes was detected, whereas in female flies a total number 11924 genes was detected. 3735 genes are significantly higher expressed in males than in females, and 7101 genes are significantly lower expressed (Fig. 1 b)).

**Fig. 2:**
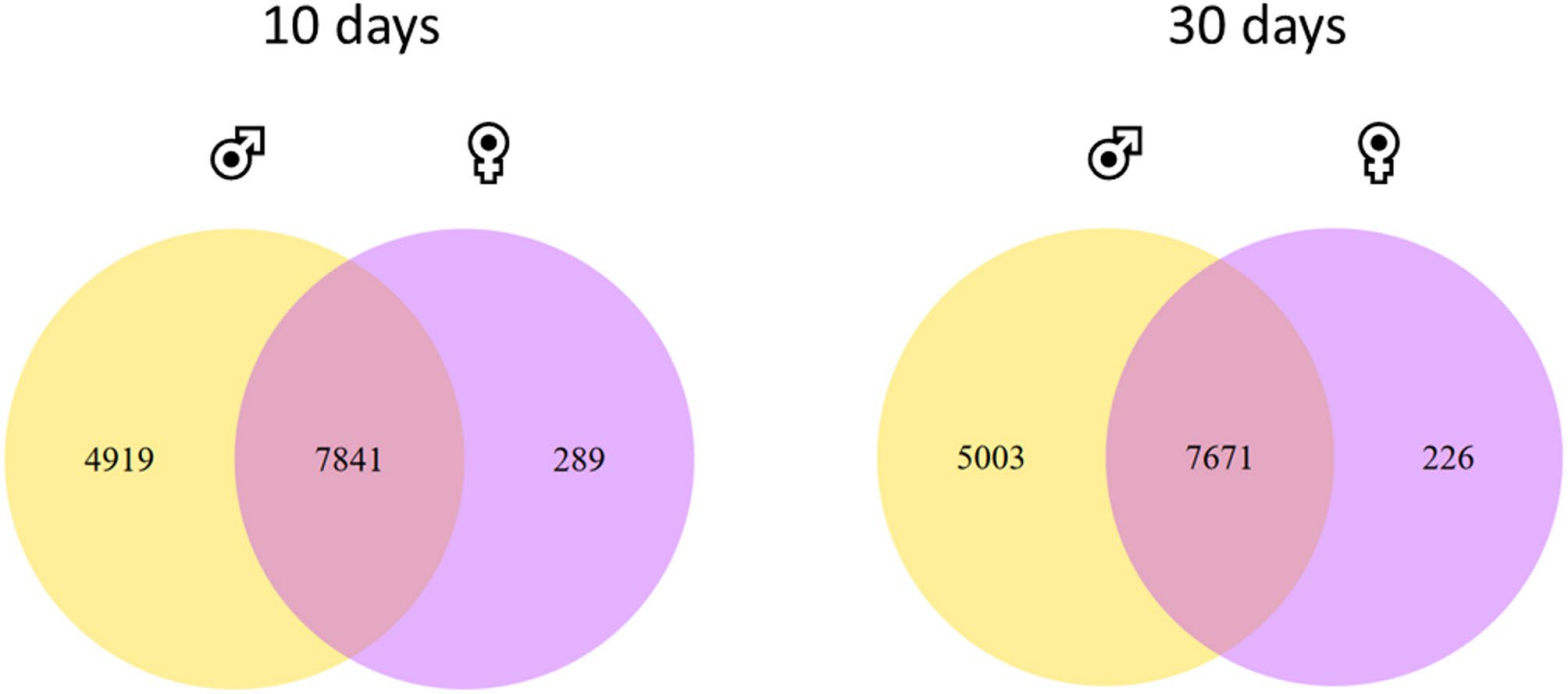
Venn diagrams showing genes expressed exclusively in males, females, or both sexes. Yellow circles represent genes exclusively expressed in males, purple circles represent genes exclusively expressed in females, and the overlay of both represents genes expressed in both sexes.

Overlay of expressed genes in males and females revealed that 10-day-old flies shared 7841 genes (Fig. 2). Male flies expressed 4919 genes that females did not express. Among these genes were different serotonin receptors, accessory gland proteins, but also different antimicrobial peptides (AMPs): *Attacin A*, *B*, *C* (*AttA*, *AttB*, *AttC*), *Cecropin A1*, *A2*, *C* (*CecA1*, *CecA2*, *CecC*), *Diptericin A* (*DiptA*), *Drosomycin-like 1*, *4* (*Drsl1*, *Drsl4*), different cytochrome P450 oxidases and *Fatp2* (*Fatty acid transport protein 2*). In female flies 289 specific genes are expressed. These include genes responsible for female reproduction (e.g. genes encoding for chorion-specific proteins or *femcoat*).

**Fig. 3:**
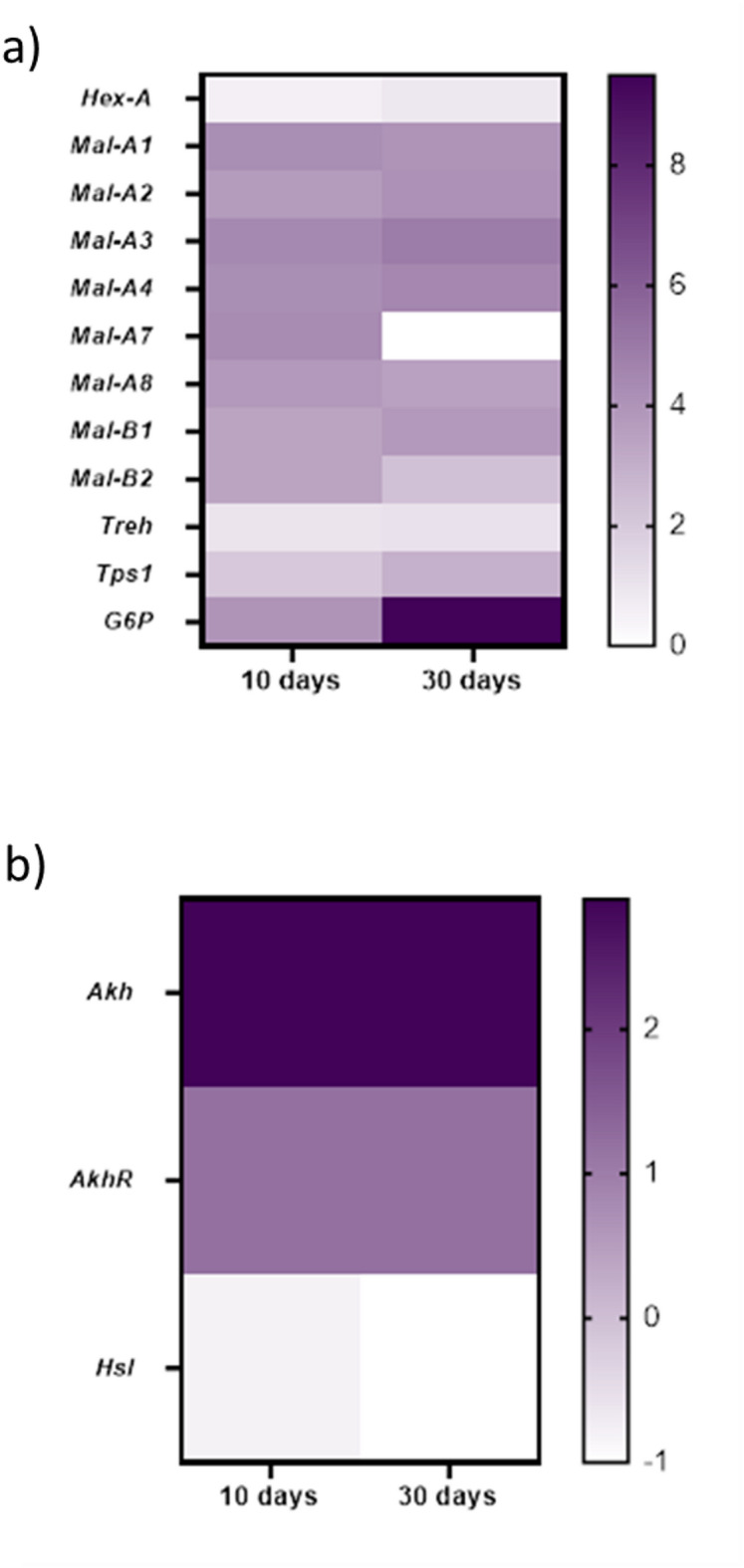
Heatmap visualizing log2 fold changes in mRNA expression levels based on FPKM values. The darker the color, the higher the mRNA expression levels in male flies compared to female flies. *Hex-A*: *Hexokinase A*; *Mal-A1/A2/A3/A4/A7/A8/B1/B2*: *Maltase* ; *A1/A2/A3/A4/A7/A8/B1/B2. Treh*: *Trehalase; Tps1*: *Trehalose-6-phosphat synthase 1*; *G6P*: *Glucose-6-phosphatase*; *Akh*: *Adipokinetic hormone*; *AkhR*: *Adipokinetic hormone receptor*; *Hsl*: *Hormone-sensitive lipase*.

In 30-day-old flies, the number of shared genes was similar (7671 genes are expressed in both sexes). There were 5003 (e.g. *AttD*, *CecB*, *Drsl3*, *Drsl4*, *Drsl5*, *Fatp2*) male-specific genes and 226 female-specific genes expressed. These genes included different chorion proteins and the vitelline membrane. When comparing different maltase encoding genes, male flies showed higher expression levels of these genes including *Maltase A1*, *A2*, *A3*, *A4*, *A7*, *A8* (*Mal-A1*, *Mal-A2*, *Mal-A3*, *Mal-A4*, *Mal-A7*, *Mal-A8*), as well as *Maltase B1* and *B2 (Mal-B1*, *Mal-B2*). This also applies to 10-day-old flies and 30-day-old flies (Fig. 3 a)). Maltases are important enzymes for the digestion of starch, hydrolyzing the disaccharide maltose into two molecules of glucose. In addition, *Hexokinase A* (*Hex-A*), *Trehalase* (*Treh*), *Trehalose-6-phosphate synthase 1* (*Tps1*), as well as *Glucose-6-phosphatase* (*G6P*) were shown to be expressed at higher levels in male flies. This is also the case for 10-day- and 30-day-old flies. However, not only genes involved in carbohydrate metabolism were expressed differentially, but also genes involved in lipid metabolism. In particular, the expression levels of the glucagon-like *Adipokinetic hormone* (*Akh*) and its receptor *Adipokinetic hormone receptor* (*AkhR*) were significantly higher expressed in males than in females, which was true for 10-day- and 30-day-old flies, while the expression level of *Hormone-sensitive lipase* (*Hsl*) was higher only in females (in 10-day- and 30-day-old flies.) (Fig. 3 b)).

**Fig. 4:**
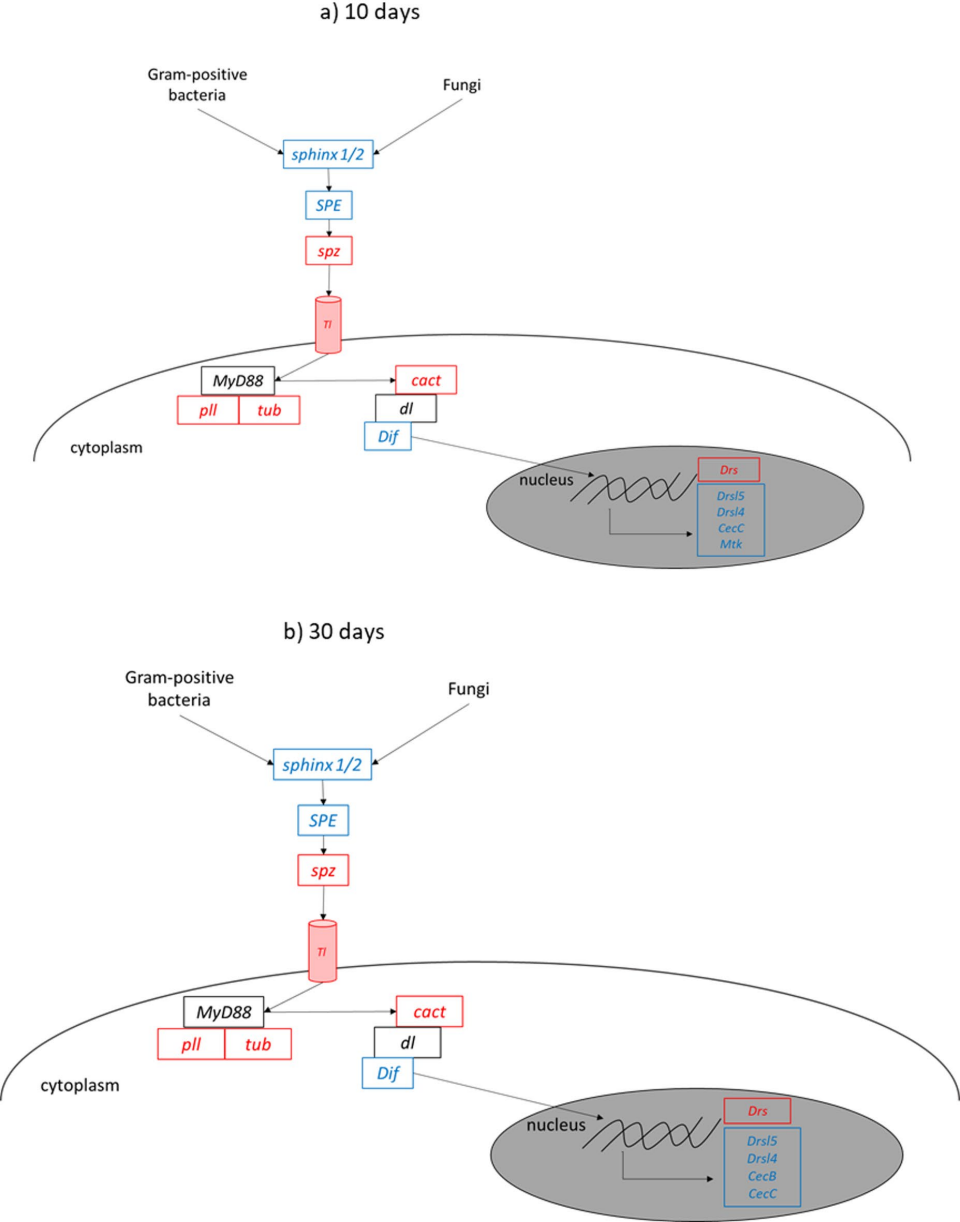
Schematic view of differentially expressed genes of toll signaling associated proteins in 10-day-old (**a**)) and 30-day-old (**b**)) flies. Genes highly expressed in female flies are depicted in red, genes highly expressed in male flies are depicted in blue, genes that do not show different expression are depicted in black. *SPE*: *Spatzle-Processing Enzyme*; *spz*: *spatzle*; *Tl*: *Toll* ;, *pll*: *pelle*, *tub*: *tube*, *cact*: *cactus*, *d*l: *dorsal*; *Dif*: *Dorsal-related immunity factor*; *Drs*: *Drosomycin*; *Drsl5/4*: *Drosomycin-like 5/4*, *CecC*: *CecropinC*; *Mtk: Metchnikowin*.

To investigate whether there are sex differences in the innate immune system, the expression levels of genes that orchestrate toll signaling were compared. Figure 4 a) presents a schematic view of toll signaling in 10-day-old flies. *sphinx1/2*, *Spatzle-Processing Enzyme* (*SPE*), *Dorsal-related immunity factor* (*Dif)* and different AMPs (*Drsl5/4*, *CecA1*, *CecC* and *Metchnikowin (Mtk)*) were higher expressed in males, whereas *spatzle* (*spz*), *Toll (Tl)*, *pelle (pll)*, *tube (tub)*, *cactus* (cact) and *Drosomycin* (*Drs*) were higher expressed in females. In 30-day-old flies, the sex specific differences seemed to be similar, except that in 30-day- old female flies *Drs* and *Mtk* are not higher expressed than in 30-day-old male flies (Fig. 4 b)).

**Fig. 5:**
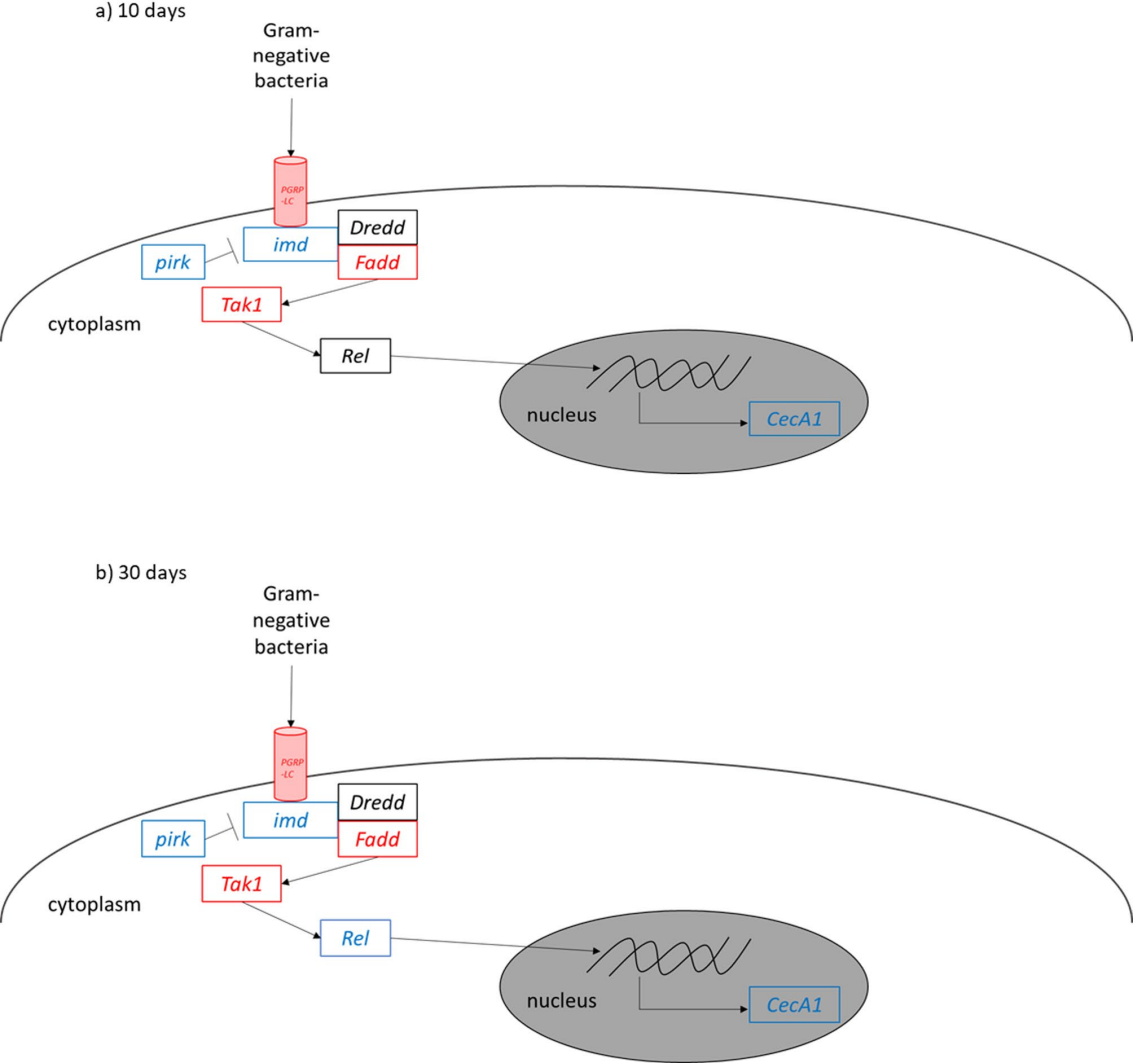
Schematic view of differentially expressed genes of the Imd pathway in 10-day-old (**a**)) and 30-day-old (**b**)) flies. Genes highly expressed in females are depicted in red, genes highly expressed in males are depicted in blue, genes that do not show different expression are depicted in black. *PGRP-LC*: *PGN-recognition protein LC*; *pirk: poor Imd response upon knock-in; imd*: *immune deficiency*; *Dredd*: *Death-related ced-3*; *Fadd*: *Fas-associated death domain*; *Tak1*: *TGFβ-activated kinase*; *Rel*: *Relish*; *CecA1*: *CecropinA1*.

In addition to toll signaling, Imd signaling is the second key regulator of immunity in *D. melanogaster*. Therefore, the expression levels of different proteins involved in this pathway were analyzed. In 10-day-old flies, the *PGN-recognition protein LC* (*PGRP-LC*), *Fas-associated death domain* (*Fadd*) and *TGFβ-activated kinase* (*Tak1*) were higher expressed in female flies compared to male flies (Fig. 5 a)), while in male flies *imd*, its inhibitor *poor Imd response upon knock-in* (*pirk*) and the AMP *CecA1* were higher expressed than in female flies. In 30-day- old flies, the sex-specific differences in the gene expression levels were similar to those in 10-day-old flies. In addition, the gene expression level of the NF-κB transcription factor *Relish* (*Rel*) was higher in male flies compared to female flies (Fig. 5 b)).

**Fig. 6:**
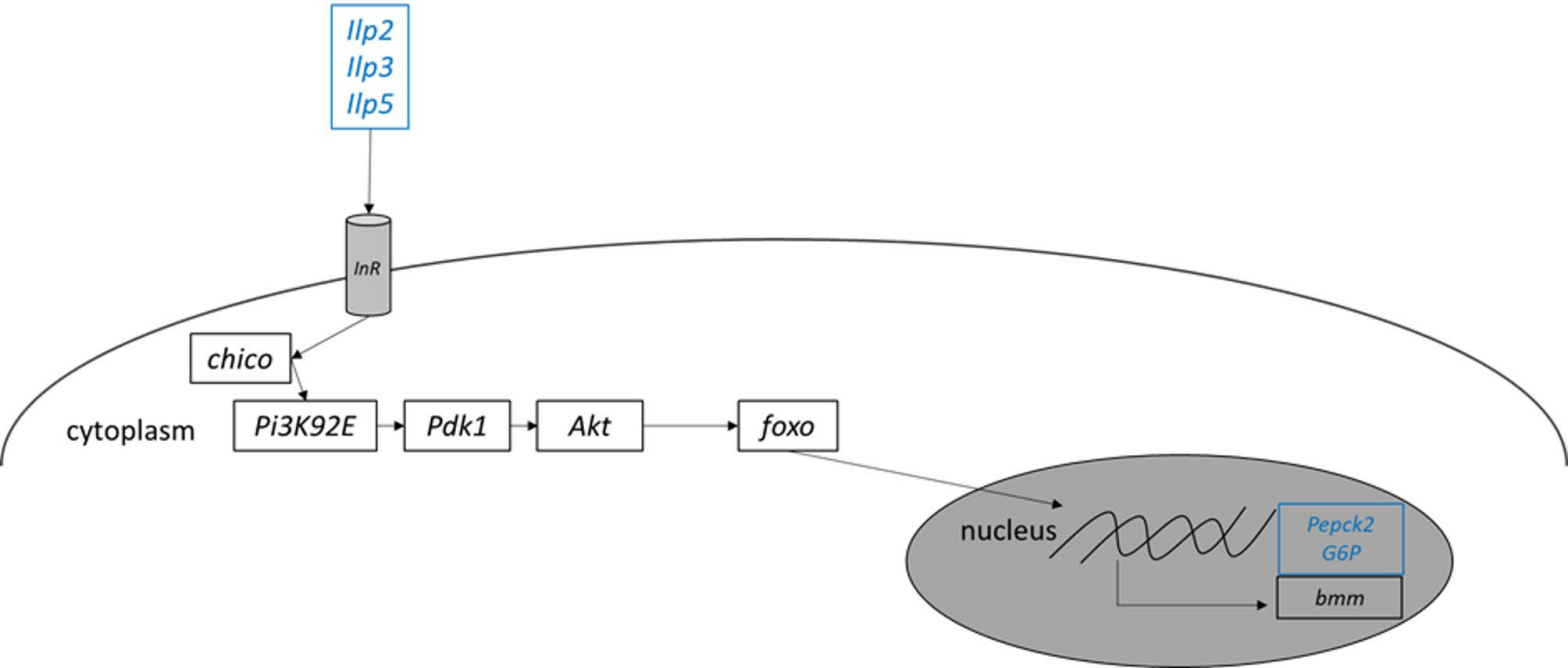
Schematic view of differences in gene expression of insulin signaling in 10-day-old and 30-day-old flies. Genes that are highly expressed in females are depicted in red, genes that are highly expressed in males are depicted in blue, genes that show no different expression are depicted in black. *Ilp2/3/5*: *Insulin-like peptide 2/3/5*; *InR*: *Insulin-like receptor*; *Pi3K92E*: *phosphoinositid-3-kinase 92E*; *Pdk1: Phosphoinositide-dependent kinase 1*; *Akt*: *Akt kinase;* foxo: *forkhead box*,* sub-group O* ,*Pepck2*: *Phosphoenolpyruvate carboxy kinase 2*; *G6P*: *Flucose-6-Phosphatase*; *bmm*: *brummer.*

Figure 6 represents schematic views of insulin signaling of 10-day-old and 30-day-old flies *D. melanogaster*. At both ages, different *Insulin-like peptides* (*Ilp2*,* 3* and *5*), *phosphoenolpyruvate carboxy kinase* 2 (*Pepck2*), and *G6P* were higher expressed in male flies. The expression levels of *Insulin-like receptor* (*InR*) and *brummer* (*bmm*) were not significantly different.

**Fig. 7:**
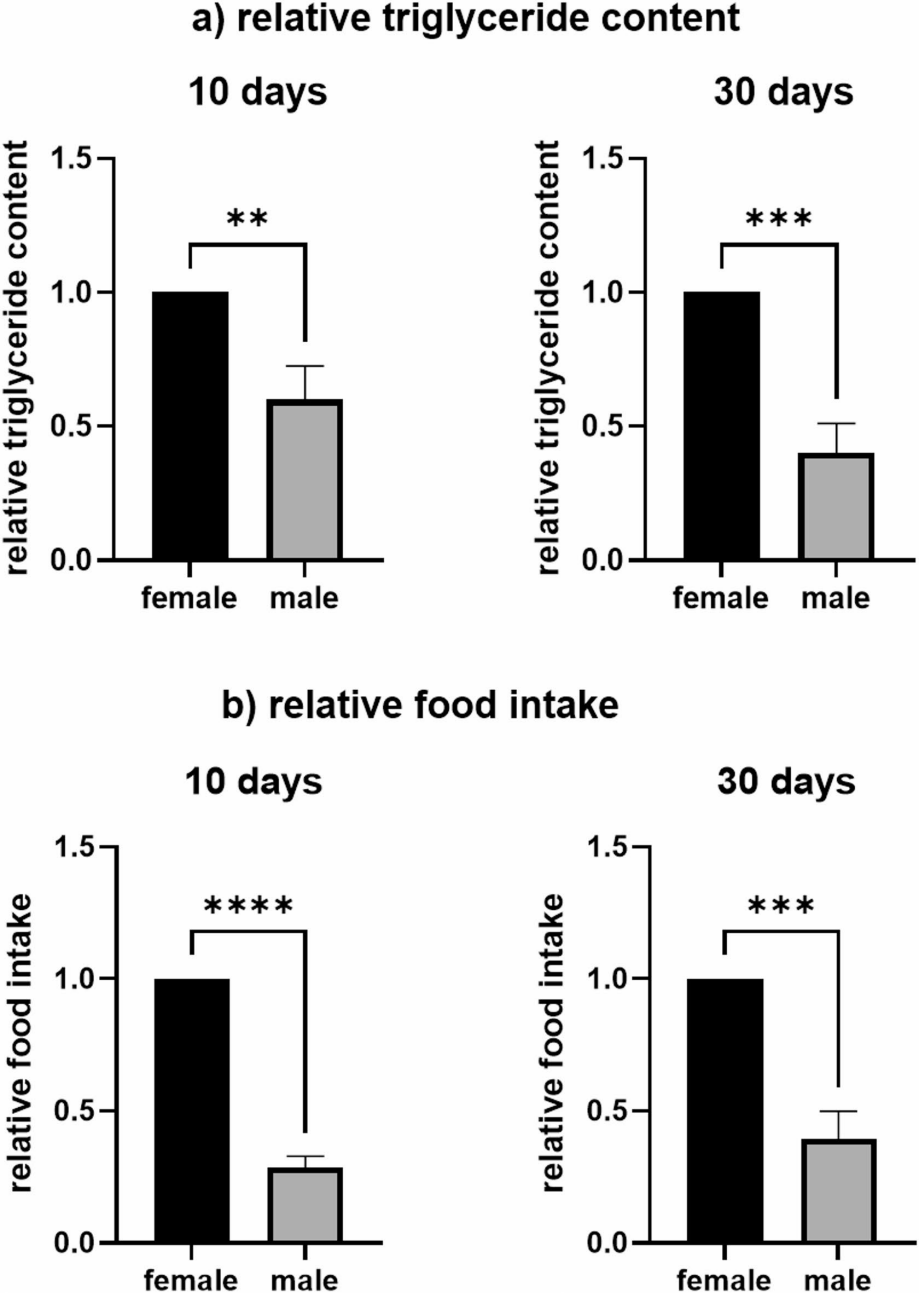
Relative triglyceride content of the whole body (**a**) of 10-day and 30-day-old female and male flies, relative food intake (**b**) of 10-day and 30-day-old female and male flies. *n* = 3. Unpaired t-test, ** *p* ≤ 0.01; ***p ≤ 0.001.

In order to analyze, if differences in gene expression result in physiological differences, we measured the triglyceride content of the whole fly body, as well as the food intake of the flies. As depicted in Fig. 7 a) the relative triglyceride content was significantly higher in females compared to males, which is true for 10-day and 30-day-old flies. The food intake also differed between both sexes: female flies had a significantly higher food intake than male flies while there was no difference between 10-day and 30-day-old flies (Fig. 7 b)).

**Fig. 8:**
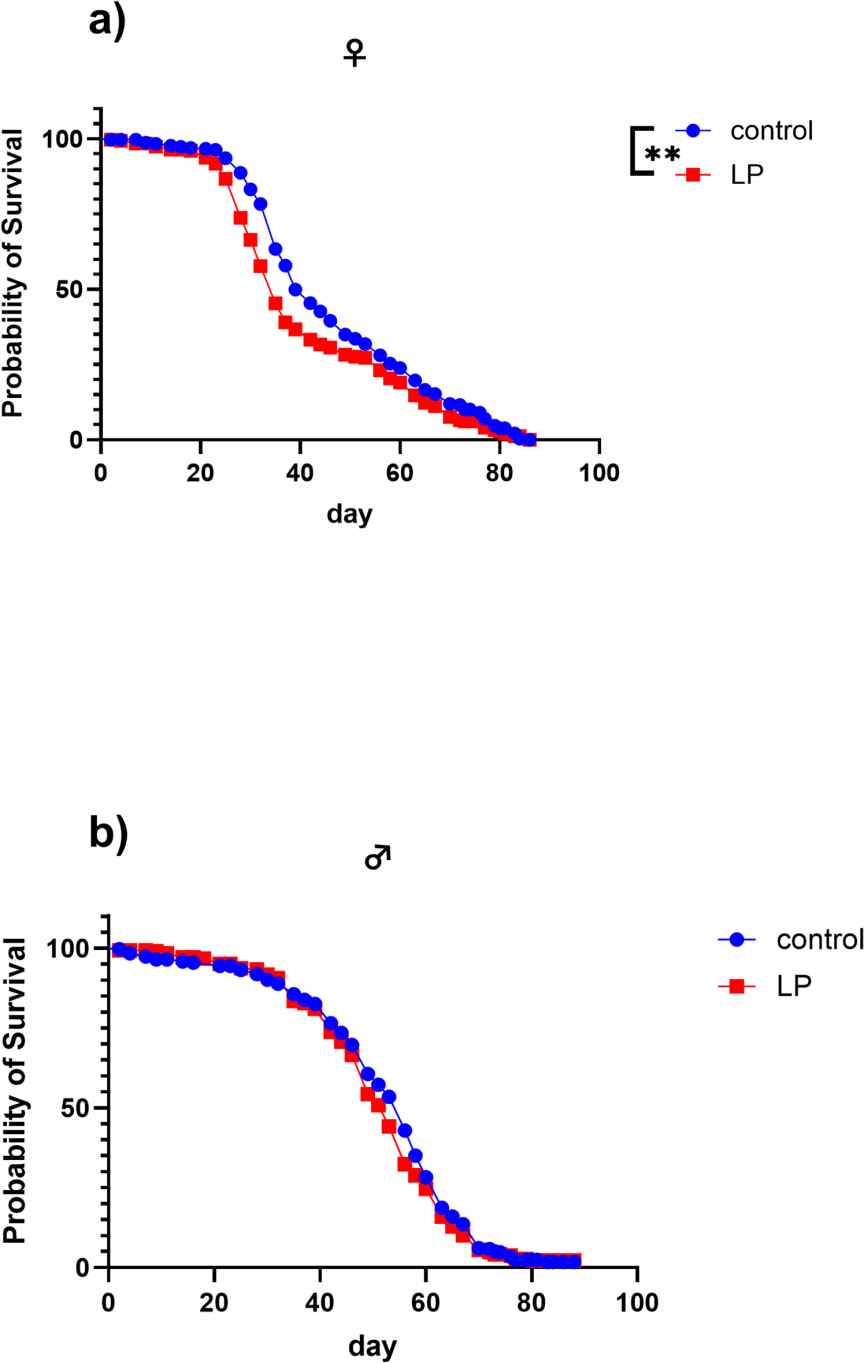
Survival curves of uninfected (control) and with *Leuconostoc pseudomesenteroides* (LP) infected female (**a**) and male (**b**) flies. Survival curves were compared with a Kaplan–Meier approach tested for significant differences by applying a log-rank test. n=4. ** *p* ≤ 0.01.

To test if the observed differences in male and female flies in the expression levels of immunity related genes are also physiologically relevant, we infected flies with *Leuconostoc pseudomesenteroides* (LP) and monitored the survival rates. Compared to male flies, female flies had a significantly shorter lifespan when exposed to the pathogen (Fig. 8).

## Discussion

The immune system of *D. melanogaster* is similar to the human immune system in its cellular and humoral components but lacks an adaptive immune system. Following pathogenic infection several effective innate immune responses are activated resulting in the expression of AMPs such as Diptericins, Metchnikowin, Defensins, Cecropins, Drosocins and Attacins^24^. The expression of these peptides is controlled by the homologs of NF-κB transcription factors, activated by toll and Imd signaling^25^. Toll signaling plays a crucial role in the immunity of both *D. melanogaster* and humans. In *D. melanogaster*, gram-positive bacteria and fungi activate toll signaling, which results in the production of AMPs (compare Fig. 4). Detailed description of toll signaling is reviewed for example in^26,27^. The present study revealed sex-specific differences in toll signaling. The *spz* activator *SPE* and the upstream *sphingx1/2* as well as the transcription factor *Dif* and different AMPs have been expressed higher in male flies. In contrast the *Tl*, its activator *spz* and the inhibitor of Dif/dl *cact* were higher expressed in female flies. This led us to hypothesize that male and female flies react differently to infections with gram-positive bacteria and fungi, which is supported by several studies^28,29^. Female flies are more likely to die from an infection with the fungus *Beauveria bassiana* than male flies, a difference that disappears in *spz* mutants^30^. The same is true for infection with the gram-negative bacterium *Providencia rettgeri*^31^. In our infection model using the fly pathogenic bacterium LP (gram-positive), only females showed a significantly reduced lifespan, supporting a possible sex-specific difference in toll pathway-mediated responses. However, further studies using pathway-specific mutants are needed to validate this hypothesis and establish a functional link between gene expression and immune competence. The reason for the difference in gene expression levels remains unclear. Given the central role of toll(like) signaling in *D. melanogaster* and in the human immune system, *D. melanogaster* could serve as a model for studying sex-specific differences in immune responses. This is particularly relevant for drug research, where understanding sex-based variations in gene expression may help to develop more targeted nutritional or therapeutic interventions by identifying molecular pathways that respond differently in males and females, thereby enabling personalized strategies to improve treatment efficacy and metabolic health.

Whereas toll signaling is among others activated by gram-positive bacteria, Imd pathway responds mainly to gram-negative bacteria through peptidoglycan (PGN) recognition^24^. PGN binds to specific recognition proteins (PGRPs), of which *D. melanogaster* possesses 19—compared to only four in mammals. Binding of PGN results in recruiting Imd following a signaling cascade which activates the transcription of AMPs like Diptericins and Cecropins^32^. Detailed description of Imd signaling is reviewed for example in^32–34^. Our study identified sex-specific differences in the Imd signaling pathway. The PGN recognition protein *PGRP-LC*, *imd*, *Rel*, *Dpt* and *CecA1* were higher expressed in male flies, while *Fadd* and *Tak1* showed higher expression levels in female flies. This is partly consistent with the results of^35^ showing higher expression level of *Dpt* in uninfected males compared to uninfected females, and a male-specific upregulation following *Pectobacterium carotovorum* subsp. *carotovorum* (a bacterium that activates Imd signaling and the expression of AMPs) infection. Additionally, our group previously observed that *P. carotovorum* infection shortened the lifespan of female but not male flies, likely due to the stronger activation of Imd signaling in males^28^. A study conducted by Vincent and Dionne in 2021^36^ supports our findings, since they observed higher expression levels of different AMPs in male flies compared to female flies following an infection with *E. coli*, bacteria that activate the Imd pathway. However, while these data suggest stronger Imd activity in males, this hypothesis needs further validation. Given the evolutionary conservation of innate immunity, *D. melanogaster* could serve as a valuable model for exploring sex-specific immune responses. This may have implications for drug research, particularly in understanding sex-based differences in immune modulation and antimicrobial therapies. But further studies are needed to confirm these hypotheses.

Not only toll signaling is a highly conserved pathway across the animal kingdom, but so is insulin signaling. Insulin/insulin-like growth factor signaling is important for development, behavior and metabolism (reviewed in for example^37–39^). *D. melanogaster* expresses eight insulin-like peptides (named as dILPs), whereas humans express one insulin and two insulin-like growth factors. In *D. melanogaster* dILP2, 3 and 5 are expressed in insulin producing cells in the brain^40^, whereas other dILPs are expressed in the fat body and the gut. In brief, insulin activates anabolic processes, while the absence of insulin promotes catabolic processes (such as gluconeogenesis). Metabolic diseases including DMT2, exhibit sex-specific differences in prevalence and severity, yet the underlying mechanisms remain unclear. Estrogen is hypothesized to play a role, but genetics may also be a key factor^10^. Recently *D. melanogaster* has been often used as a model organism for diet-related metabolic disorders^22^, making it an ideal system to investigate these sex-specific effects. We found that male flies had higher expression levels of *Ilps* (*Ilp2*, *Ilp3*, *Ilp5*), *Pepck2* and *G6P*, which is consistent with the expression atlas FlyAtlas2^41^. Interestingly, dILPs produced in the brain (*Ilp2*, *Ilp3*, *Ilp5*) were upregulated in males, while those from the fat body and gut showed no sex differences. These findings align with previous studies showing higher *Ilp3* expression in male larvae, while female larvae secreted higher levels of dILP2 protein^42^. However, these results are only partially comparable because (a) insulin signaling differs between larvae and adult flies and (b) a change in mRNA levels is not necessarily reflected in the protein levels of a gene. The higher expression of *Ilp3* and *Ilp5* in adult males should be considered when using *D. melanogaster* to study diet-induced metabolic disorders. However, this hypothesis remains to be proven. Prior to the onset of insulin action, ingested complex carbohydrates such as starch initially need to be hydrolyzed by α-amylase resulting in maltose molecules, which are further hydrolyzed to glucose by maltases. In *D. melanogaster*, ten genes encode for maltose-degrading enzymes^43^. In the present study, we showed that different maltases are higher expressed in male flies, which is consistent with FlyAtlas2^41^ and the results of^44^, who showed that the protein levels of Mal-A1, Mal-A3, Mal-A7 and Hex-A are more abundant in male than in female intestines. These results suggest that male and female flies digest food, especially ingested carbohydrates, in different ways.

Although numerous studies have explored diet-induced metabolic disorders in *D. melanogaster*, most focused either on larvae^14,45–48^, on only one sex^22,23,49^, the sex of the flies was not mentioned, or males and females were analyzed in one sample^50^. Usually, metabolic disorders in *D. melanogaster* are induced by chronic feeding of a high carbohydrate diet (especially a high sucrose diet) or a high fat diet (for example coconut oil). Given the observed sex differences in digestive enzyme expression and insulin signaling, males and females likely respond differently to dietary challenges. Future studies using *D. melanogaster* as a model should account for these sex-specific metabolic responses to ensure accurate conclusions in nutrition and drug research.

Mammalian females store about 10% more fat than males, while female insects can store up to four times more fat than male insects^51^. In insects, these fat reserves likely support egg production, as reduced fat mass decreases fecundity in females^52^, while excess fat storage lowers reproductive success in male insects^53^. Additionally, female insects mobilize stored fat less efficiently than males during food scarcity^53^ suggesting a differential regulation of lipid metabolism in male and female *D. melanogaster*. The Akh signaling pathway (which is functional homologous to glucagon signaling in humans) is a crucial regulator of lipid metabolism (reviewed in^54,55^). In brief, active Akh signaling leads to increased fat mobilization and decreased fat storage. Consistent with previous findings^16^, our study confirmed higher expression of *Akh* and its receptor (*AkhR*) in male flies. Also FlyAtlas2 data show a male-biased expression of these genes^41^. Moreover, our phenotypic data revealed that females had higher triglyceride levels, supporting the data on gene expression and suggesting a sex-specific regulation of lipid metabolism. Additionally, sex differences in food intake were observed, potentially contributing to differences in fat storage and energy balance. We found no sex differences in *bmm* expression, differing from earlier research where virgin males exhibited higher *bmm* mRNA levels than females^53^. This discrepancy may be due to differences in mating status, as our study analyzed mated flies. It is documented, that mating changes expression levels of different genes^56^. However, even with similar *bmm* expression, sex-specific differences in protein function remain possible. The expression level of *Hsl* in our study was higher in female flies compared to male flies, which was in accordance with other studies suggesting that lipid mobilization differs by sex. This supports the suitability of *D. melanogaster* to study sex-specific aspects of diet-induced metabolic disorders, though functional analyses are necessary to validate these transcript-based conclusions. Although the observed gene expression patterns are consistent with known sex-specific differences in immunity and metabolism, definitive conclusions about pathway activity or physiological function cannot be drawn as the present study is mainly based on transcriptomic data. Future studies should include for example protein-level validations and the use of mutant strains. It should also be taken into account, that the laboratory strain w^1118^ is not a real wild type strain, leading to differences for example in gene expression, physiology and behavior^57^. As the present results are limited to mated flies these results are not necessarily true for virgin flies^56^. Despite these limitations, our study could serve as a starting point for more specific investigations.

Overall, the fruit fly *D. melanogaster* could serve as a cost-effective and easy-to-handle model organism to investigate sex specific differences in detail. Our findings highlight significant variations between male and female flies in immune signaling pathways (toll and Imd), insulin signaling, and lipid metabolism. Males show higher expression levels of genes involved in immune signaling pathways, higher expression levels of insulin-like peptides (*Ilp2*, *Ilp3*, *Ilp5*), and increased expression levels of *Akh* and *AkhR*. Because those pathways are homologous to corresponding pathways in humans this model could help analyzing the sex-specific effects of dietary interventions or drugs, ultimately leading to a better understanding of sex-specific interconnections and improving the development of more effective, sex-specific medical treatments.

## Materials and methods

### Drosophila melanogaster w^1118^ stocks

In the present study, all experiments were performed with w^1118^ *Drosophila melanogaster* (Bloomington Drosophila Stock Center, Indiana, USA; #5905). Flies were reared on 10% Caltech medium (CT) under standard conditions in a climate chamber (HPP 1018, Memmert, Schwalbach, Germany) at 25 °C, 60% relative humidity, and a 12-h day/night cycle. For experiments, 3-day-old (to ensure, all flies were mated) age-matched flies from synchronized eggs were immobilized on ice and separated according to their sex. Female and male flies were transferred to separate vials containing a diet with 10% sugar (SY10 medium).

### Media

Caltech medium (CT) consists of 5.5% dextrose, 3.0% sucrose (Carl Roth, Karlsruhe, Germany), 6.0% corn meal, 2.5% inactive dry yeast, 1.0% agar, 0.3% Tegosept (Kisker, Steinfurt, Germany), and 0.3% propionic acid (Carl Roth, Karlsruhe, Germany). SY10 medium comprises 10% sucrose, 10% inactive dry yeast, 2.0% agar 0.3% Tegosept (Kisker, Steinfurt, Germany), and 0.3% propionic acid (Carl Roth, Karlsruhe, Germany).

### RNA isolation and sequencing

RNA isolation was performed with the Quick-RNA Tissue/Insect kit (Zymo Research, Freiburg, Germany) according to the manufacturer’s instructions. For each sample, 10 flies were homogenized in extraction buffer in a Tissue Lyzer II (Qiagen, Hilden, Germany). Following RNA isolation, the purity was detected photometrically (260/280nm) in a UV-Mini 1240 UV-VIS Spectrophotometer (Shimadzu, Duisburg, Germany). Samples with a 260/280 nm ratio lower than 1.7 were excluded from further analysis. For each condition, RNA was isolated from three independent experiments. RNA sample library preparation, RNA-seq, data collection, bioinformatic and statistical analysis was performed by Novogene Co., Ltd (Beijing, China). In brief, RNA quantity, quality and integrity were determined using an Agilent 5400 Fragment Analyzer System (Agilent Technologies). mRNA was isolated from total RNA by Poly(A) capture. After library preparation a 150 bp paired-end sequencing strategy was used for sequencing. Raw RNA-seq reads were mapped to the *D. melanogaster* reference genome, and expression levels were quantified using FPKM (fragements per kilobase of transcript per million mapped reads). Differentially expressed genes were identified by DESeq2 analysis, applying the Wald test and adjusted p-values using the Benjamini-Hochberg method. Genes with adjusted *p*-value < 0.05 and |log2(FoldChange)| > 1 were considered as differentially expressed. No outliers were removed and all biological replicates were included in the final analysis. Batch effects were assessed using principal component analysis (PCA) and hierarchical clustering; no significant batch-related biases were observed. Raw data for the discussed genes can be found in the Supplementary Information. Three independent biological replicates were generated, each consisting of three technical replicates. To create volcano plots and Venn diagrams, NovoMagic was used, to create heatmaps GraphPad Prism 10 (GraphPad Software, Boston, USA) was used.

### Triglyceride content

For the measurement of the fly’s triglyceride content, 25 age-matched flies were maintained on SY10 for 10 or 30 days. Then, flies were weighed and frozen at -80 °C until further use. For measurements, 5 flies were homogenized in 250 µl PBS with 1% triton X in a Tissue Lyzer II (Qiagen, Hilden, Germany). Following, samples were centrifuged at 5000 g for 10 min. The supernatant was transferred into a fresh vial and stored at -20 °C until further use. For the detection of the triglyceride content a Trigylceride-Kit (Dialab, Neudorf, Austria) was used, following the manufacturer’s instructions. For calculation of the triglyceride content values were initially normalized to the fly weight and finally to the control group. To compare the results an unpaired t-test was performed using GraphPad Prism 10 (GraphPad Software, Boston, USA) and significance was accepted at *p* < 0.05. Three independent biological replicates were generated, each consisting of three technical replicates.

### Bacterial strains and cultivation

For infection assays, the fly-pathogenic bacterial strain LP, kindly provided by Dr. Kwang-Zin Lee from the Fraunhofer Institute for Molecular Biology and Applied Ecology (IME, Giessen, Germany), was used. LP was grown in MRS broth (Carl Roth) and cultivated under aerobic conditions in a shaking incubator (B. Braun Biotech International, Melsungen, Germany) at 29 °C overnight. For all experimental infections, bacterial cultures were used in their stationary growth phase.

### Oral infection with *Leuconostoc pseudomesenteroides* (LP) and survival assays

For oral infections, overnight cultures of LP were diluted with a sterile 100 mM sucrose solution to an optical density (OD) of 1. 1 ml of the bacterial suspension or the sterile sucrose solution (used as control) was applied to three layers of cellulose paper lining the bottom of each vial. Age-matched 10-day-old male and female *D. melanogaster* w^1118^, derived from synchronized egg collections, were sorted according to their sex, introduced into the vials (25 flies per vial) and maintained under standard conditions for 18 h. After 18 h of infection, the flies were transferred to fresh vials, containing SY10 medium. Every second day, the flies were transferred to fresh SY10 medium containing vials while dead flies were counted and removed. Survival curves were compared with the Kaplan–Meier approach followed by a log-rank test to test for significant differences which were accepted at *p* < 0.05. Four independent biological replicates were generated, each consisting of three technical replicates.

### Food intake

To assess the food intake, 25 age-matched male and female flies per vial were maintained on SY10 for 10 or 30 days. Subsequently, the flies were transferred to new vials containing food supplemented with 0.2% sulforhodamine B (Carl Roth, Karlsruhe, Germany). After 8 h of feeding, flies were weighed and frozen at − 80 °C until further processing. For analysis, 20 flies per sample were homogenized in 200 µl PBS containing 1% Triton X using a Tissue Lyzer II (Qiagen, Hilden, Germany) and centrifuged at 5000 g for 5 min. The supernatant was then transferred to a 96-well plate, and the fluorescence signal was detected (ex/em: 565/586 nm). For calculation of the food intake, the fluorescence signal was initially normalized to the weight of the flies and this value subsequently normalized to the control group. To test for significant differences between the two groups an unpaired t-test was performed using GraphPad Prism 10 (GraphPad Software, Boston, USA) while significance was accepted at *p* < 0.05. Three independent biological replicates were generated, each consisting of three technical replicates.

## Electronic supplementary material

Below is the link to the electronic supplementary material.

Supplementary Material 1

Supplementary Material 2

## Data Availability

The datasets generated and analysed during the current study are available in Gene Expression Omnibus (GEO), accession number GSE293388.
