# Supplementary material for: Drosophila melanogaster as a model organism to investigate sex specific differences
Source: Sci Rep. 2025 Jun 4;15:19648. doi: 10.1038/s41598-025-04497-0 (PMC12137727; doi:10.1038/s41598-025-04497-0)
Supplement: Supplementary file 1 — Supplementary Material 1 [file 41598_2025_4497_MOESM1_ESM.docx]

Supplementary Information

**S 1: Heatmap visualizing log2 fold changes in mRNA expression levels.** The darker the color, the higher the mRNA expression levels in male flies compared to female flies. SPE: Spatzle-Processing Enzyme; spz: spatzle; Tl: Toll ;, pll: pelle, tub: tube, cact: cactus, dl: dorsal; Dif: Dorsal-related immunity factor; Drs: Drosomycin; Drsl5/4: Drosomycin-like 5/4, CecC: CecropinC; Mtk: Metchnikowin; PGRP-LC: PGN-recognition protein LC; pirk: poor Imd response upon knock-in; imd: immune deficiency; Dredd: Death-related ced-3; Fadd: Fas-associated death domain; Tak1: TGFβ-activated kinase; Rel: Relish; CecA1: CecropinA1.

**S 2: Heatmap visualizing log2 fold changes in mRNA expression levels.** The darker the color, the higher the mRNA expression levels in male flies compared to female flies. Ilp2/3/5: Insulin-like peptide 2/3/5;,Pepck2: Phosphoenolpyruvate carboxy kinase 2; G6P: Flucose-6-Phosphatase.
